# Computational modeling for rational design of novel phenoxy tacrine derivatives targeting Alzheimer’s disease

**DOI:** 10.1371/journal.pone.0343723

**Published:** 2026-03-05

**Authors:** Mohamed El Fadili, Mohammed Er-rajy, Somdutt Mujwar, Abdelouahid Samadi, Samir Chtita, Menana Elhallaoui

**Affiliations:** 1 LIMAS Laboratory, Faculty of Sciences Dhar El Mahraz, Sidi Mohamed Ben Abdellah University, Fez, Morocco; 2 Chitkara College of Pharmacy, Chitkara University, Rajpura, Punjab, India; 3 Department of Chemistry, College of Science, United Arab Emirates University, Al Ain, United Arab Emirates; 4 Laboratory of Analytical and Molecular Chemistry, Faculty of Sciences Ben M’Sik, Hassan II University of Casablanca, Sidi Othman, Casablanca, Morocco; Inonu University, Faculty of Pharmacy, TÜRKIYE

## Abstract

Alzheimer’s is the leading factor behind dementia, producing steady impairments in memory, cognitive reasoning, behavioral, and social interactions. This scientific study investigates thirty-two phenoxy tacrine (PhO-THA) derivatives through an integrated computational modeling to identify potential therapeutic candidates. 3D-QSAR models were developed using comparative molecular similarity indices analysis and comparative molecular field analysis, which were subjected to rigorous internal and external validation to establish a robust quantitative relationship between molecular interaction fields and cytotoxic activities. Based on these validated structural insights, fourteen new compounds (D1-D14) were designed. Comprehensive molecular docking and molecular dynamics (MD) simulations, coupled with ADME-Tox profiling, were used to evaluate their pharmacological potential. Our results highlight four specific compounds (D9-D12) that exhibit favorable pharmacokinetic properties and a high safety profile, making them promising candidates for future drug development. D9 was selected for MD simulations due to its lower cytotoxic activity (pIC_50_ of 3.50), which is comparable to the reference THA drug (pIC_50_ of 3.52). The results demonstrated exceptional thermodynamic stability for D9 upon complexation with the NMDA receptor (PDB ID: 5EWJ) over a 100 ns simulation time.

## 1. Introduction

According to the most recent 2019 statistics, approximately 50 million people worldwide suffer from Alzheimer’s disease (AD) as one of the global health issues that requires urgent intervention to save a large population segment from potentially devastating consequences [1].

Tacrine drug, namely 1,2,3,4-tetrahydro-9-aminoacridine (THA), is commonly used in medicinal chemistry as the first Food and Drug Administration (FDA)-approved therapy for AD, due to its good reactivity and multi-target effect, despite its withdrawal for a long time due to an undesirable hepatotoxicity for the human body. Nowadays, the scientific research community is directed toward discovering new tacrine derivatives, considering that the THA drug is the main nucleus that would reveal effective candidate therapies against Alzheimer’s disease [2–5]. N-methyl-d-aspartate receptors (NMDARs) have gained enormous importance in the treatment of neurodegenerative disorders that affect the central nervous system (CNS), especially schizophrenia, neuropathic pain, and AD [6–8]. However, the candidate drugs that target NMDARs are often associated with undesirable side effects of psychosis and hepatotoxicity. Acetylcholinesterase (AChE) inhibition is the second biological target for AD treatment, which leads to the relief of symptoms provided by cholinergic activity. For this reason, the combination of AChE inhibition and NMDA antagonism is a multi-target approach to design a variety of drug candidates for AD treatment [9,10]. More recently, thirty-two phenoxy tacrine (PhO-THA) derivatives were synthesized based on the 7-phenoxytacrine (7-PhO-THA) compound [11], as a successfully synthesized product, a dual-action agent with *in vivo* neuroprotective activity, inhibiting both AChE and NMDA receptors in a balanced manner [11].

In the present study, we examined a structural family of thirty-two PhO-THA derivatives, which were previously developed to reduce NMDAR cytotoxicity and minimize the hepatotoxicity commonly associated with THA-mediated chemical compounds. Our objective was to design new potent derivatives with favorable physicochemical and pharmacokinetic profiles targeting the active sites of NMDAR with high thermodynamic stability. To achieve this, we used a comprehensive computer-aided drug design (CADD) strategy, starting with the generation of robust three-dimensional structural-activity relationship (3D-QSAR) models using comparative molecular field analysis (CoMFA) and comparative molecular similarity index analysis (CoMSIA) to identify structural features responsible for cytotoxic activity [12,13]. Next, we performed computational analysis of absorption, distribution, metabolism, excretion, and toxicity (ADME-Tox) of the designed compounds [14–16]. Then, we subjected the most active designed derivatives to molecular docking with the GluN1-GluN2B subunit of the NMDARs. Finally, we performed high-resolution molecular dynamics (MD) simulations over 100 nanoseconds [15,17] to evaluate the structural stability and binding persistence of the designed PhO-THA ligands within the NMDA targeted receptors.

## 2. Materials and methods

### 2.1. 3D QSAR study

**Fig 1:**
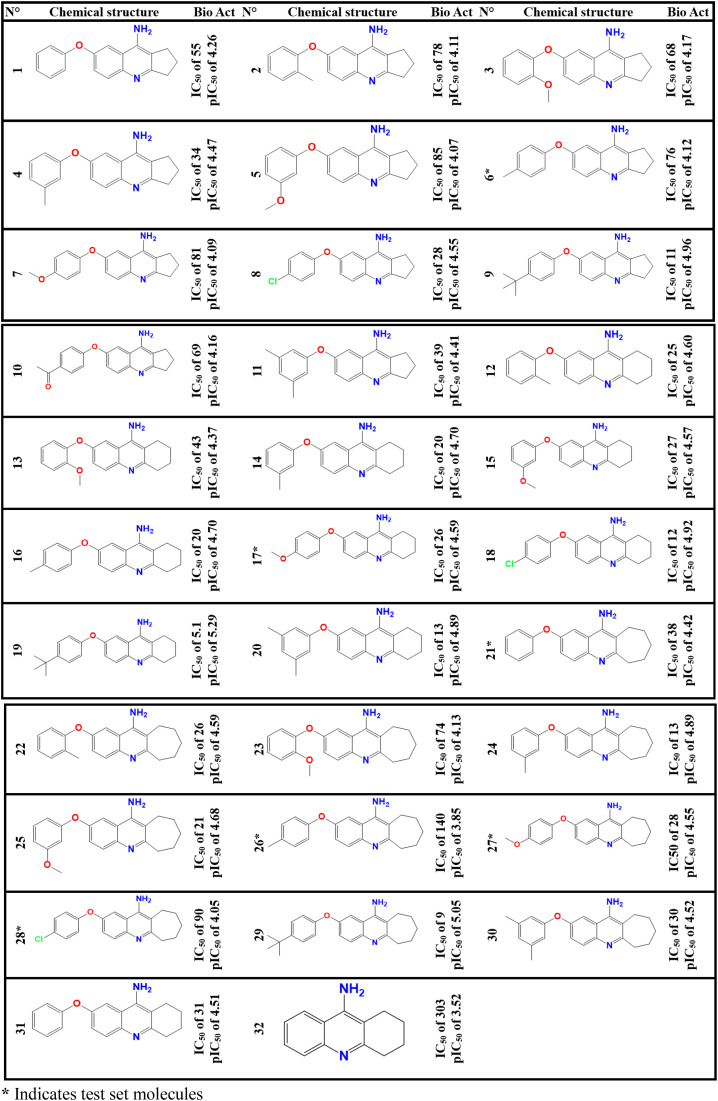
Examined molecules with biological activities of IC_50_ (µmol) and pIC_50_ order.

**Fig 2:**
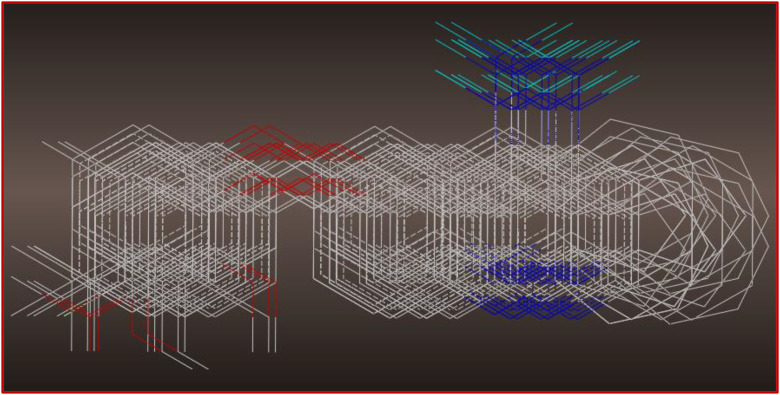
Superposition of 31 PhO-THA derivatives on the most active compound (THA drug).

To examine the quantitative relationship between thirty-two PhO-THA derivatives and their cytotoxic activities of pIC_50_ order (Fig 1), a 3D QSAR method was carried out using SYBYL.X.2.0 software, in which the chemical structure of each active molecule was firstly optimized by minimizing the energies in kcal.mol^-1^ to guarantee the molecular stability for each geometrical conformation, after adding the Gasteiger-Hückel charges and structural optimization was carried out with the Powell-gradient method, where the convergence parameter was set to 0.001 kcal·mol ⁻ ¹·Å ⁻ ¹ with 10000 iterations as the maximum, while using the Tripos-force fields. In the second stage, the optimized molecules were aligned on the THA drug (C32) as the less cytotoxic compound (template) and the common nucleus of the studied database (Fig 2). This is a crucial step to ensure the reliability and robustness of classical CoMSIA and CoMFA models, respectively [18–20]. Thereafter, the optimized data was separated into two subsets, a training set containing twenty-six molecules (80% of statistical data) and the test assembly comprising six molecules (20% of statistical data), in which the test and training set molecules were obtained through dozens of randomized trials, each of which we have searched for each randomization a training set that will subsequently be internally validated by Cross-validation using the “leave-one-out” strategy (CV-LOO) such that the model generated will be strongly validated using external validation technique. Ultimately, the six following molecules (6*, 17*, 21*, 26*, 27*, and 28*) were singled out as an appropriate test set, so the remaining ones were chosen as a training set.

### 2.2. Pharmaceutical similarity and *in silico* ADME-Tox predictions

Among the most urgent and important tasks in recent pharmaceutical science is to produce safe drugs with the highest efficacy while saving time and minimizing effort and financial cost [14]. For this reason, a computer-assisted drug design known as the CADD technique has gained enormous importance in the pharmaceutical sciences. In the present stage, the designed compounds’ pharmacokinetic features, including ADME-Tox, were properly predicted using PKCSM and Swiss ADME servers [21].

### 2.3. Molecular docking simulations

Molecular docking is a technique widely used in drug discovery to detect inhibition mechanisms with minimal binding affinity in kcal/mol, which was executed using Discovery Studio 2021 and AutoDock.4.2 programs [22–24], where all four designed compounds, labeled D9, D10, D11, D12, and the most active compound (C32; THA drug), were chosen to be docked towards N-methyl-D-aspartate receptor encoded by 5EWJ.pdb, which corresponds to the crystal structure of the NMDAR amino-terminal domains of the GluN1 and GluN2B subunits, obtained by X-ray diffraction at a resolution of 2.77 Å (https://www.rcsb.org/structure/5EWJ). This targeted receptor, composed of the GluN1 and GluN2B subunits, is widely studied in neurodegenerative disease research, since its dysfunction is associated with neuronal damage. Therefore, inhibition of this ion channel was reported to be beneficial in the treatment of several neurological disorders, particularly Alzheimer’s disease [25]. For this objective, the mentioned protein was prepared by assigning Gasteiger charges, deleting water molecules (H_2_O), and co-crystallized ligands [22,23,26]. Then, D9, D10, D11, and D12 designed molecules were docked to the prepared macromolecule to be compared with the THA drug by using Autodock4.2 software [27,28], in which the grid box was centralized each time on the targeted receptor with cartesian coordinates of x = 83.161, y = 11.011, and z = −37.326 with a nuclear spacing of 0.375 Å. In the second stage, the Discovery-Studio-2021 software was employed to guess the produced interactions in 2D and 3D [29,30].

### 2.4. Molecular dynamics simulations

MD simulation with a detailed resolution was performed to investigate the thermodynamic stability of the resulting intermolecular contacts throughout the MD simulation using Desmond, a package of the LLC-Schrödinger program. The input files of the MDs were selected as the Output files of molecular docking [31,32]. MD simulations were carried out using the OPLS-2005 force field, validated for proteins, nucleic acids, and small molecules. Before simulation, the complexes were prepared by assigning bond orders from the CCD database, adding missing hydrogens, forming disulfide bonds, and defining zero-order bonds to metal ions, followed by structural optimization with PROPKA at pH 7.0. Water molecules were modeled to ensure proper solvation and maintain system stability, supporting accurate energy calculations and realistic protein-ligand interactions. Simulations were performed for 100 ns, producing around 1000 frames with 1.2 energy [33]. The simulation temperature was kept at 330 K with a Nose-Hoover chain thermostat, and pressure was regulated at 1 atm using a Martyna-Tobias-Klein barostat with a 2.0 ps relaxation time and isotropic coupling. Counterions were added to neutralize the system, and 0.15 M Na⁺ and Cl⁻ salt was included to replicate physiological conditions [7,17,34].

## 3. Results and discussions

### 3.1. 3D QSAR study

By using the partial least square (PLS) regression, a group of seventeen 3D-QSAR models shown in Table 1, were properly generated after calculating various molecular interaction fields, inclusive of Electrostatic (E), Steric (S), and Hydrophobic (H), in addition to Acceptor (A), and Donor (D) fields of Hydrogen bonds. In principle, Steric and Electrostatic fields are classically implicated in CoMFA model, so that Electrostatic, Steric, Hydrophobic, Donor, and Acceptor, of Hydrogen bonds are encompassed in CoMSIA models. Statistically, to obtain a robust, applicable, internally and externally validated 3D QSAR model, Q^2^cv coefficient estimated by LOO option and the non-CV coefficient (R^2^) must be superior to 0.6 and 0.7, respectively. The computed value of Fisher (F) must be superior to the critical value, and the standard error of estimation (SEE) must be minimal for an optimum number of main components (ONC). Finally, the external (EV) coefficient must exceed the 0.6 threshold [12]. All statistical values were calculated for CoMFA model and all 16 of CoMSIA models, working on the same training set of 26 molecules to generate the 3D QSAR model each time and validate it internally using the CV-LOO technique, then validate it externally for the same test set selected previously (6*, 17*, 21*, 26*, 27* and 28*) using the external validation technique.

**Table 1:** Statistical evaluation of CoMSIA and CoMFA models using different combinations of molecular fields.

All possible 3D-QSAR Models	Fractions of molecular interactions fields					*Q* ^*2*^_*cv*_	*R* ^*2*^	*SEE*	*F*	*ONC*	*R* ^*2*^ *pr*
	S	E	H	D	A						
CoMSIA/SEA	0.143	0.807	–	–	0.050	0.650	0.867	0.152	34.230	4	0.371
CoMSIA/SEH	0.087	0.588	0.325	–	–	0.717	0.832	0.163	57.135	2	0.694
CoMSIA/SED	0.108	0.809	–	0.084	–	0.678	0.798	0.183	29.044	3	0.522
CoMSIA/SHD	0.186	–	0.663	0.152	–	0.662	0.799	0.179	45.751	2	0.771
CoMSIA/SHA	0.182	–	0.566	–	0.252	0.653	0.786	0.185	42.215	2	0.776
CoMSIA/SDA	0.608	–	–	0.129	0.263	0.410	0.692	0.232	11.772	4	0.008
CoMSIA/EHA	–	0.537	0.393	–	0.070	0.713	0.860	0.157	32.129	4	0.514
CoMSIA/EHD	–	0.555	0.395	0.049	–	0.701	0.850	0.158	41.619	3	0.574
CoMSIA/EDA	–	0.822	–	0.074	0.104	0.612	0.813	0.181	22.797	4	0.420
CoMSIA/HDA	–	–	0.692	0.145	0.164	0.639	0.796	0.185	28.540	3	0.780
CoMSIA/EHDA	–	0.513	0.397	0.040	0.050	0.713	0.859	0.157	32.055	4	0.526
CoMSIA/SHDA	0.184	–	0.614	0.093	0.108	0.643	0.804	0.181	30.114	3	0.715
CoMSIA/SEDA	0.103	0.717	–	0.064	0.116	0.601	0.792	0.186	27.940	3	0.283
CoMSIA/SEHA	0.084	0.498	0.359	–	0.059	0.699	0.866	0.153	33.790	4	0.476
CoMSIA/SEHD	0.071	0.529	0.349	0.052	–	0.709	0.847	0.160	40.693	3	0.600
CoMSIA/SEHDA	0.072	0.488	0.335	0.049	0.056	0.700	0.854	0.156	42.921	3	0.471
CoMFA	0.536	0.464	–	–	–	0.712	0.883	0.143	39.466	4	0.601

**Fig 3:**
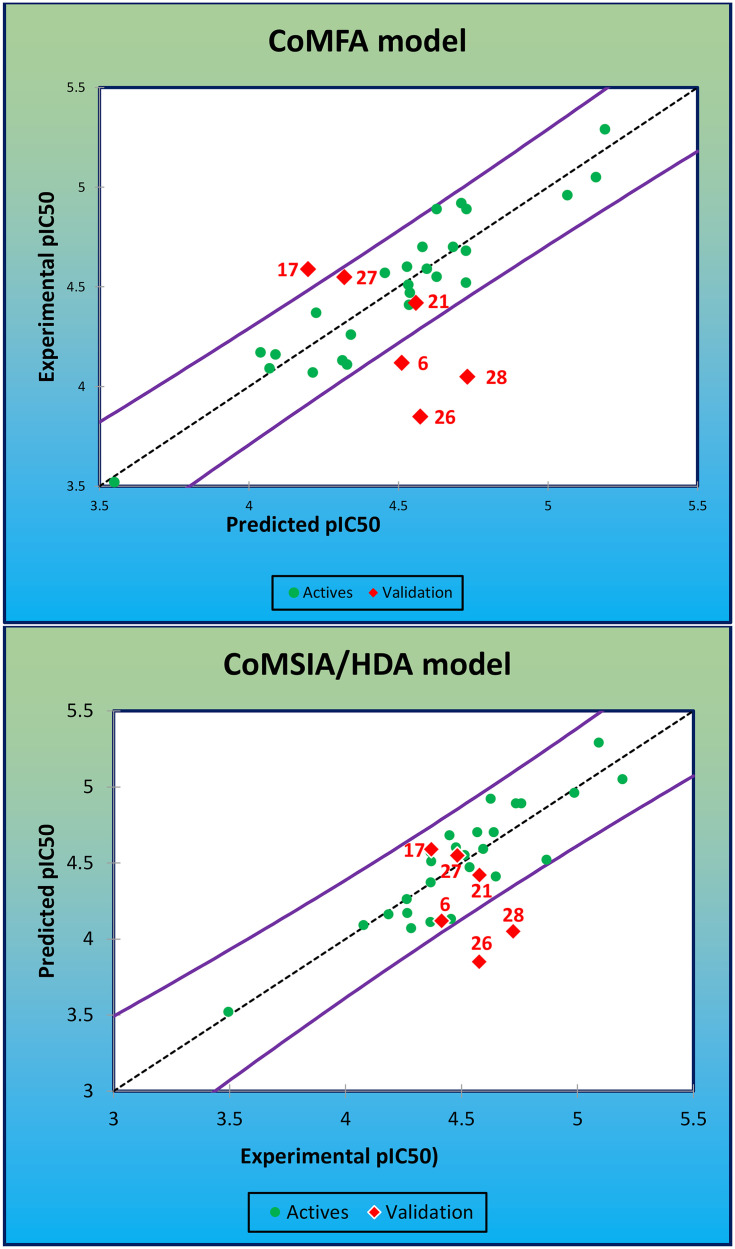
Produced correlations between the Exp and Pred cytotoxic activities by CoMFA and CoMSIA/HDA, respectively.

The obtained findings indicate that the CoMFA model given by the following statistical criteria: *Q*^*2*^_*cv*_ of 0.712, *R*^*2*^ of 0.883, *SEE* of 0.143, *Fisher* value of 39.466, and *R*^*2*^*pr* of 0.601 was strongly checked by both internal and external validation, which demonstrates that cytotoxic activity of the studied PhO-THA derivatives is strongly affected by Steric and Electrostatic molecular fields. Furthermore, among the sixteen remaining combinations, the COMSIA/HDA model was the best CoMSIA model that is strongly examined by both external and internal validation with a *Q*^*2*^_*cv*_ of 0.639, *R*^*2*^ of 0.796, *SEE* of 0.185, *Fisher* value of 28.540, and *R*^*2*^*pr* of 0.780, thereby demonstrating that H, A, and D of Hydrogen bonds have a special effect in the cytotoxic activity against Alzheimer disease. Consequently, the predicted values of the cytotoxic activities with pIC_50_ order for all 26 training molecules are presented in Table 2, using both the CoMFA and CoMSIA/HDA models. So, the expected values of pIC_50_ order for the test set including six compounds (6*−17*−21*−26*−27*−28*) are presented in Table 3, in which the correlation resulted between the predicted (Pred) and experimental (Exp) cytotoxic activities of training molecules as mentioned in green, and the test set molecules as colored in red are presented in Fig 3 for both examined 3D/QSAR models.

**Table 2:** Prediction of NMDAR-antagonizing activities using 3D/QSAR models.

N°	Exp pIC_50_	Pred pIC_50_ by CoMFA	Pred pIC_50_ by CoMSIA/HDA
C1	4.26	4.341	4.264
C10	4.16	4.089	4.185
C11	4.41	4.536	4.648
C12	4.6	4.529	4.476
C13	4.37	4.225	4.368
C14	4.7	4.683	4.64
C15	4.57	4.455	4.366
C16	4.7	4.58	4.568
C18	4.92	4.71	4.626
C19	5.29	5.191	5.092
C2	4.11	4.328	4.366
C20	4.89	4.628	4.758
C22	4.59	4.595	4.594
C23	4.13	4.313	4.455
C24	4.89	4.728	4.735
C25	4.68	4.726	4.448
C29	5.05	5.161	5.194
C3	4.17	4.039	4.267
C30	4.52	4.726	4.867
C31	4.51	4.533	4.37
C32	3.52	3.55	3.495
C4	4.47	4.538	4.535
C5	4.07	4.214	4.283
C7	4.09	4.069	4.078
C8	4.55	4.628	4.514
C9	4.96	5.065	4.987

**Table 3:** Results of EV corresponding to CoMSIA/EDA and CoMFA.

N°	Observed pIC_50_	Predicted pIC_50_ using CoMFA	Predicted pIC_50_ using CoMSIA/HDA
17*	4.59	4.197	4.369
21*	4.42	4.558	4.577
26*	3.85	4.572	4.576
27*	4.55	4.318	4.481
28*	4.05	4.730	4.722
6*	4.12	4.510	4.414

* Indicates the test set molecules.

### 3.2. Analysis of graphical representations of successfully valorized 3D QSAR models

**Fig 4:**
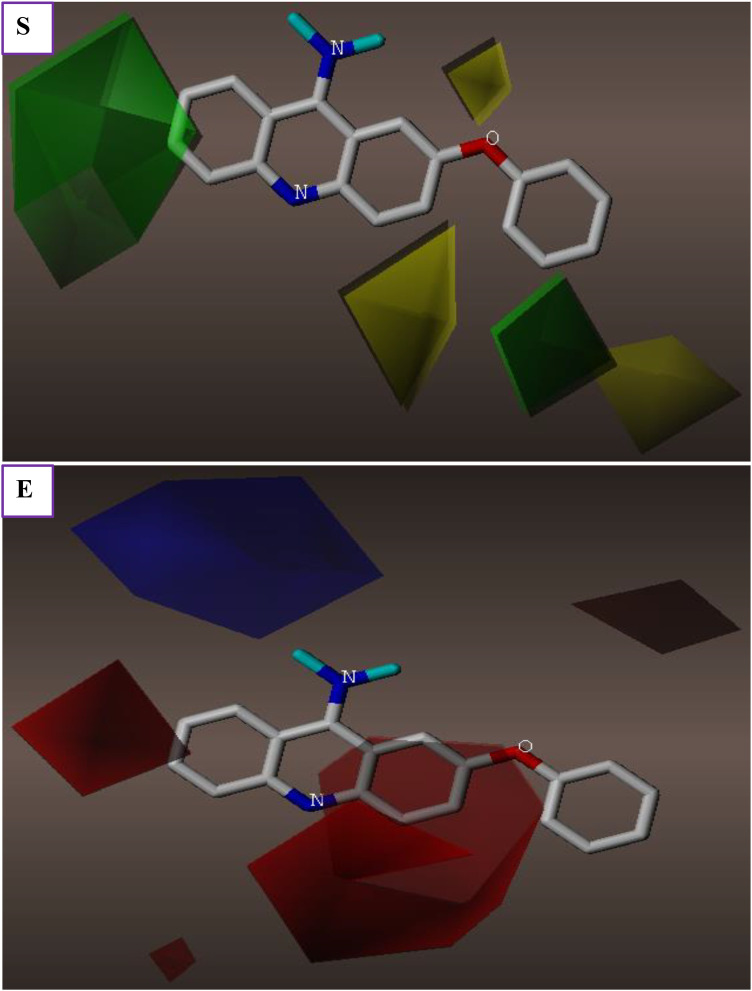
Electrostatic and Steric fields involved in CoMFA for 7-phenoxy tacrine derivative (C31). **(E)** Electrostatic fields. **(S)** Steric fields.

**Fig 5:**
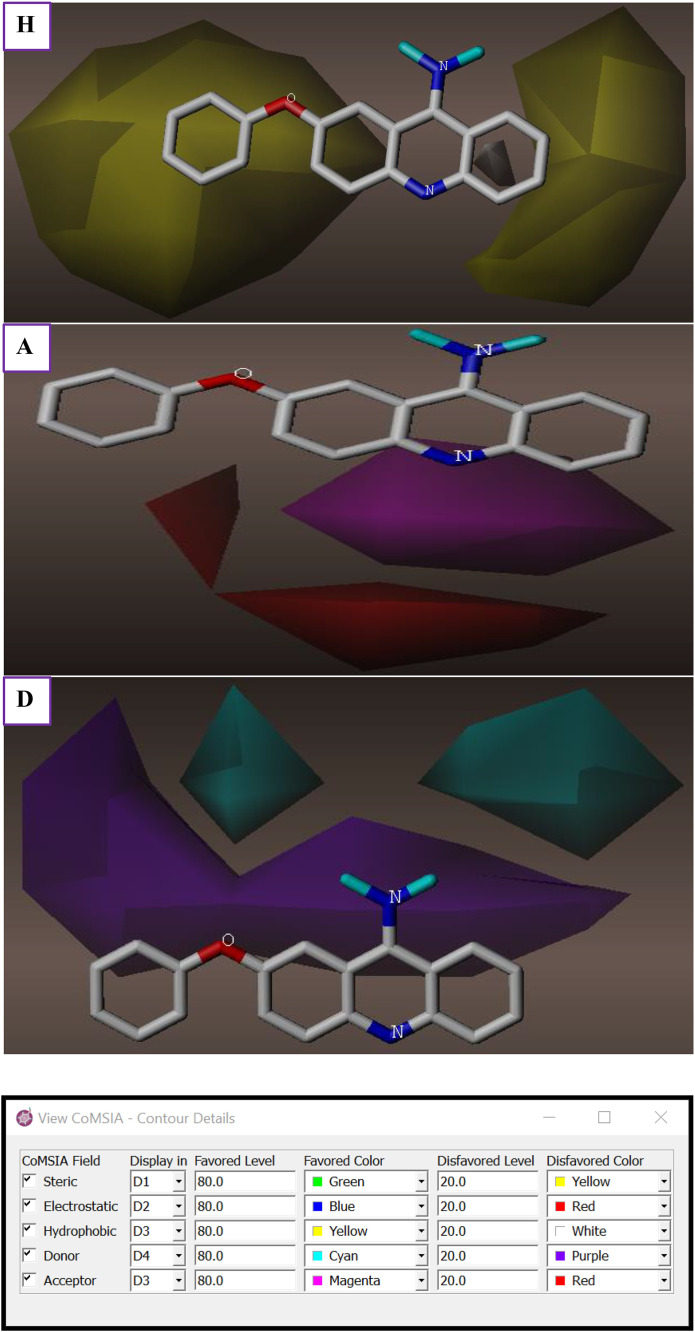
Hydrophobic, Donor, and Acceptor of H-bonds fields involved in CoMSIA/HAD for 7-phenoxy tacrine derivative (C31) (H) Hydrophobic fields. (D) Donor fields of H-bonds. (A) Acceptor fields of H-bonds.

**Fig 6:**
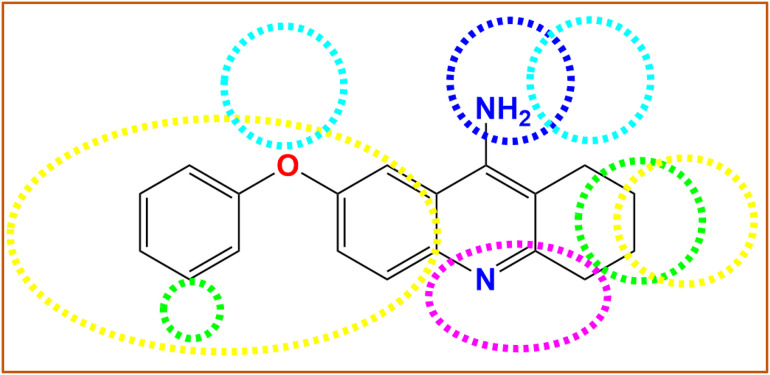
3D-QSAR contour maps showing favorable fields of S (green), E (blue), H (yellow), A (magenta), and D (cyan).

**Fig 7:**
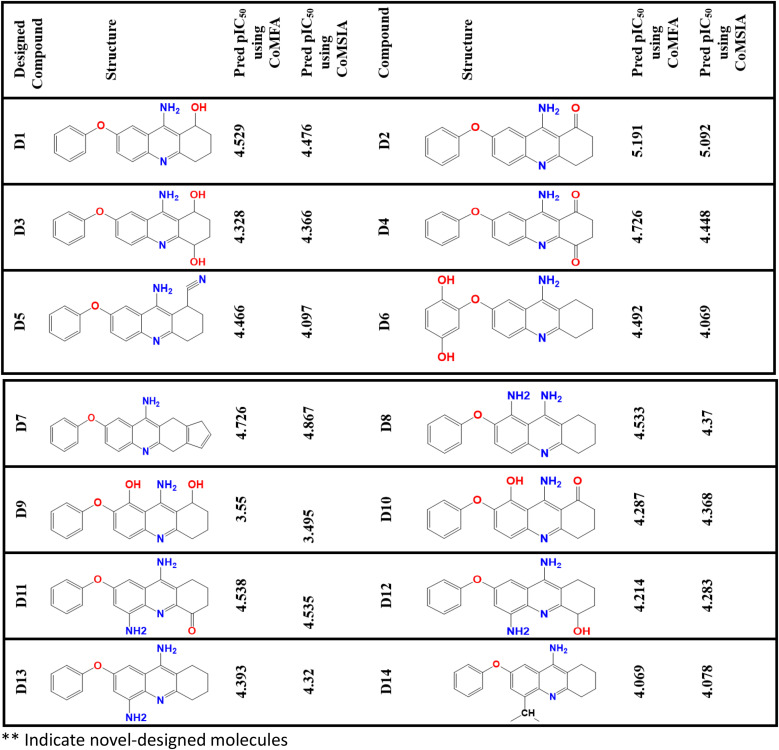
pIC_50_ predicted values of the designed compounds based on CoMSIA/HDA and CoMFA models.

3D contour plots for various domains involved in molecular interactions within CoMFA and CoMSIA/HDA models were properly employed to distinguish regions contributing positively or negatively to S, E, H, D, and A of Hydrogen bond fields that could justify the cytotoxic activity of PhO-THA derivatives (Fig 4). The obtained findings demonstrate that steric fields displayed in Fig 4S were located in the vicinity of the phenoxy group and the cyclohexane ring linked to the naphthalene core, as the green and yellow contours, which contribute respectively by 80% and 20%, show the corresponding regions and macroaggregates that can increase or decrease the cell’s cytotoxic activity. Second, the electrostatic fields shown in Fig 4E indicate that the amino group attached to the 1,2,3,4-tetrahydro acridine nucleus is the single cluster that can help to produce a strong electrostatic field while other parts of the studied molecule can adversely affect the electrostatic field, where 80% of the favorable contributions are reflected by the blue lines, and 20% of these unfavorable contributions are presented by the red lines. Regarding the CoMSIA/HDA model (Fig 5), and similarly to steric fields, 80% of Hydrophobic fields are favored by the phenoxy group and the cyclohexane ring linked to the naphthalene core as colored in yellow lines in Fig 5H. Finally, Hydrogen bond acceptor and donor fields are well-exposed around the 1,2,3,4-tetrahydro-9-aminoacridine core, in which 80% of favorable contributions of acceptor fields are presented in magenta (Fig 5A), and 80% of favorable donor field contributions are colored by cyan lines, as shown in Fig 5D. The catalytic/favorable areas of different molecular fields, including steric, electrostatic derived from the CoMFA model, more than H, A, and D of hydrogen bonds derived from CoMSIA/HDA model, demonstrate 3D structural-activity relationships (SAR) established between the most active phenoxy tacrine derivative (C31) on behalf of all other derivatives and the cytotoxic activity, as shown in Fig 6. These findings have greatly helped us discover a total of fourteen new chemical compounds of PhO-THA derivatives, whose biological activities of pIC_50_ order were predicted using both CoMSIA/HDA and CoMFA, as resulted in Fig 7.

### 3.3. Pharmacokinetics profiling

**Fig 8:**
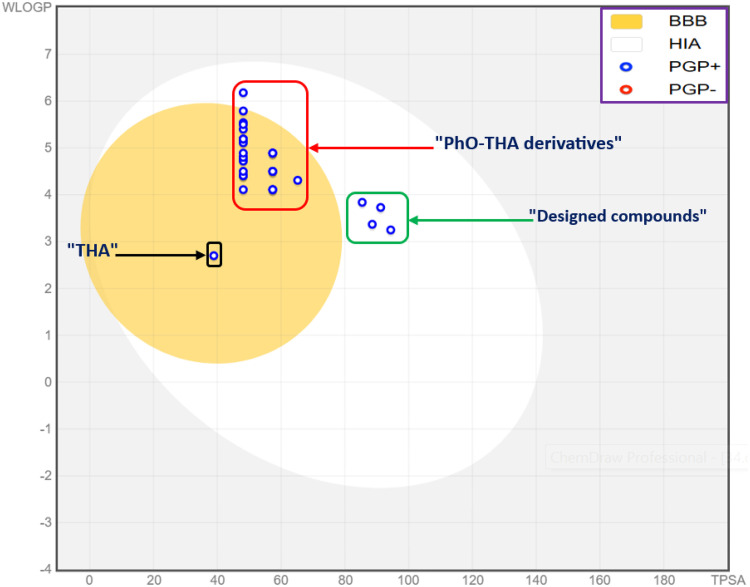
Boiled egg model for THA, PhO-THA derivatives, and novel-designed compounds.

In-silico screening of pharmacokinetic properties of ADME-Tox [28] applied to novel designed compounds reveals the toxicity of ten designed compounds, which were predicted to have positive AMES toxicity, and reveals genetic changes, making them potential rodent carcinogens. In contrast, only four molecules labeled D9, D10, D11, and D12 were predicted to be potentially safe, with an excellent ADME profile, supported by the high human intestinal absorption (IA) values above 91%, along with favorable permeabilities across CNS and BBB, potent inhibition to 1A2 and 3A4 cytochromes, as presented in Table 4. These candidate molecules were equally examined by their physicochemical features which were predicted based on Lipinski rules in addition to the violations of Egan, Ghose, Veber, and Muegge as presented in Table 5, in which all four designed compounds satisfied the violations number of Muegge, Veber, Egan, Muegge, and Ghose moreover they meet all Lipinski rules of five in which molecular weights exceed not 500g/mol, Log P less than five, topological surface area inferior than 140 A², Hydrogen bond acceptor and donor not exceed ten and five thresholds, respectively. Furthermore, the predictive model of boiled egg shows that all four designed small-molecules are part of the white part of Egan’s egg as presented in Fig 8, which confirms a predictable chance of being absorbed passively by the gastrointestinal tract in a different way than THA and synthesized PhO-THA derivatives which were expected to passively permeate across BBB due to their location in the yellow area of Egan’s egg [35]. The clearly visible gaps in Fig 8 graphically represent the success in modifying physicochemical features achieved through rational drug design. The incorporation of specific polar and non-polar functional groups significantly modulates the balance between Topological Polar Surface Area (TPSA) and WLOGP (lipophilicity), effectively moving the studied analogues away from the parent THA scaffold’s features. This results in the clear clustering, which corresponds to fundamental changes in their predicted absorption (HIA) and distribution (BBB permeability or PGP substrate likelihood) characteristics, thus confirming that the rational design strategy successfully tuned the ADME-Tox parameters.

**Table 4:** Predicted pharmacokinetic ADME-Tox features of designed PhO-THA derivatives.

N°	ADME-Tox Properties											
	A-	D-		M-							E-	T-
	IA (human)	BBB permeability	CNS permeability	CYP450							Total clearance	AMES toxicity
				Substrate			Inhibitor					
				2D6	3A4	1A2	2C19	2C9	2D6	3A4		
Unity	(%absorbed)	(Log BB)	(Log PS)	(YES/NO)							(log mL min^-1 kg-1^)	(yes/no)
Predicted values												
D1	90.868	−0.065	−1.881	No	Yes	Yes	Yes	Yes	No	Yes	0.279	Yes
D2	93.491	−0.072	−1.844	No	Yes	Yes	Yes	Yes	No	No	0.123	Yes
D3	90.194	−0.95	−2.123	No	Yes	Yes	No	Yes	No	No	0.386	Yes
D4	93.846	−0.467	−2.04	No	Yes	Yes	Yes	Yes	No	No	0.269	Yes
D5	93.553	−0.185	−1.684	No	Yes	Yes	Yes	Yes	No	No	0.33	Yes
D6	91.557	−0.902	−2.007	No	No	Yes	Yes	Yes	No	No	0.304	Yes
D7	93.89	0.013	−1.717	No	Yes	Yes	Yes	Yes	No	No	0.268	Yes
D8	94.024	0.174	−1.782	No	Yes	Yes	Yes	Yes	No	No	0.102	Yes
D9	91.246	−0.798	−2.039	No	Yes	Yes	Yes	No	No	Yes	0.104	No
D10	92.359	−0.229	−2.002	No	Yes	Yes	Yes	No	No	Yes	0.004	No
D11	92.612	−0.007	−1.959	No	No	Yes	No	Yes	No	Yes	0.339	No
D12	92.752	−0.752	−2.009	No	No	Yes	Yes	Yes	No	Yes	0.286	No
D13	93.257	0.144	−1.768	No	Yes	Yes	Yes	Yes	No	No	0.26	Yes
D14	94.884	−0.054	−0.991	No	Yes	Yes	Yes	Yes	No	Yes	0.688	Yes

** Indicate novel-designed molecules.

**Table 5:** Prediction of physicochemical properties of four designed compounds.

	TPSA	MW	LogP	n-HBA	n-HBD	Violations number				
						Lipinski	Veber	Egan	Muegge	Ghose
Rule	<140 A²	<500 D.a	≤5	<10	<5	≤2	≤2	≤2	≤2	≤2
D9	88.60	322.36	2.92	4	3	Yes	Yes	Yes	Yes	Yes
D10	85.44	320.34	2.87	4	2	Yes	Yes	Yes	Yes	Yes
D11	91.23	319.36	2.17	3	2	Yes	Yes	Yes	Yes	Yes
D12	94.39	321.37	2.38	3	3	Yes	Yes	Yes	Yes	Yes

### 3.4. Molecular docking simulations

**Fig 9:**
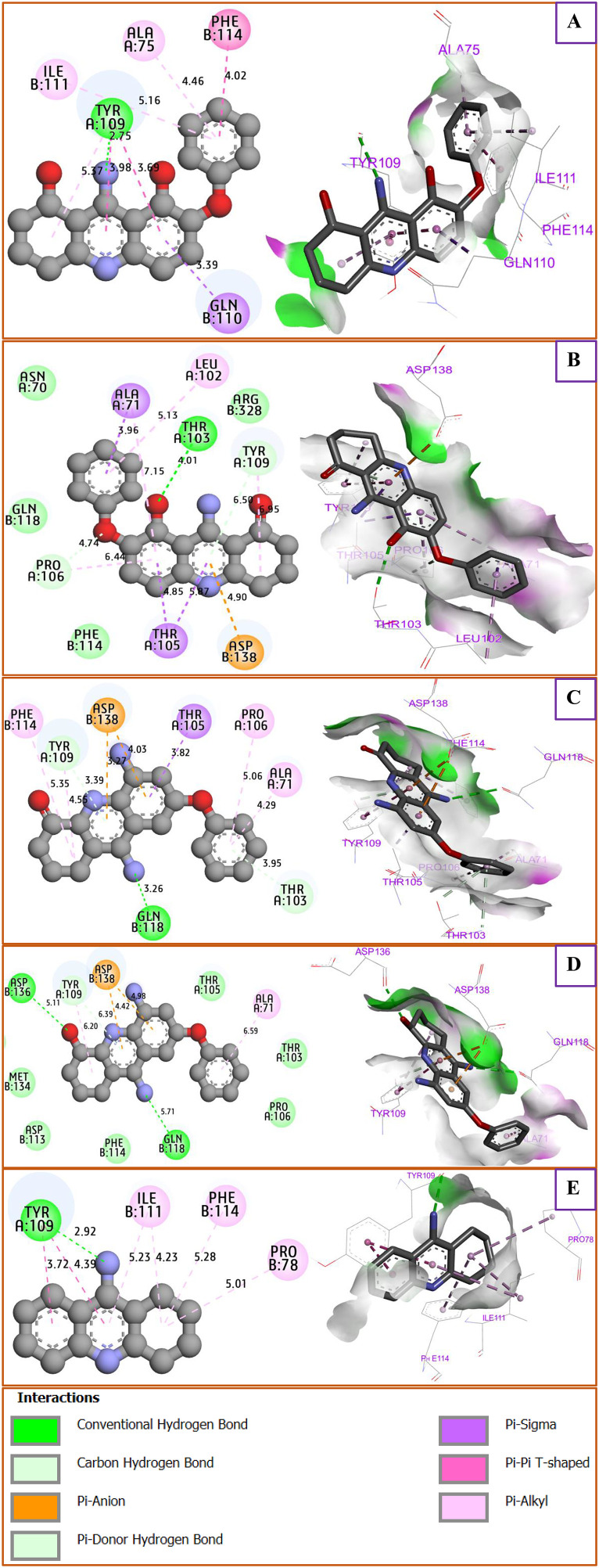
2D and 3D views showing the binding interactions of the NMDAR with THA drug (C32), D9, D10, D11, and D12 designed compounds. (A) D9 in complex with NMDAR. (B) D10 in complex with NMDAR. (C) D11 in complex with NMDAR. (D) D12 in complex with NMDAR. (E) C32 in complex with NMDAR.

All four designed compounds, labeled D9, D10, D11, and D12, which were discovered as safe candidate inhibitors with excellent physicochemical and ADME pharmacokinetic profiles, were selected for molecular docking studies targeting the NMDA receptor subunits GluN1 and GluN2B (5EWJ), then compared to the intermolecular interactions produced for the THA drug after its complexation to the same protein (Fig 9). Firstly, the molecular docking results show that the designed molecule D9 was complexed to the targeted protein of the NMDA receptor, producing several key interactions inclusive of H-bond with Tyr109 amino acid residue (A/A/R), Pi-Sigma bond fixed to Gln110 active site, Pi-Pi Stacked bond detected by Phe114 amino acid residue, in addition to Alkyl and Pi-Alkyl bonds created with Ile111 and Ala75 A/A/R, as shown in Fig 9A. Second, (D10-NMDA receptor) complex reveals the formation of H-bond detected with Thr103 A/A/R, one Pi-anion bond with Asp138 A/A/R, two Pi-Sigma bonds created with Ala71 and Thr105 A/A/Rs, more than one Pi-Alkyl bond detected with Leu102 A/A/R, more than two pi-donor H-bonds with Tyr109 and Pro106 A/A/Rs, as presented in Fig 9B. Third, the designed compounds D11 and D12 were equally docked to NMDAR, as displayed in Fig 9C and Fig 9D, respectively, revealing almost similar interactions, including one common H-bond produced with Gln118, one Pi-Sulfur bond fixed with Asp138, one Pi-donor H-bond with Tyr109 A/A/R, in addition to one Pi-Alkyl bond produced with Ala71 A/A/R. For comparison, the produced interactions are almost the same intermolecular contacts as those detected towards the (THA drug-in NMDA receptor) complex shown in Fig 9E, simulated with a bonding energy of −7.76 kcal.mol^-1^, revealing a common chemical bond with Tyr109 A/A/R, more than common Ile111 A/A/R interacted with D9 compound, one common Phe114 interaction with D11 designed compound. Furthermore, all designed compounds D10, D11, and D12 were complexed to NMDA receptors with very close energies in kcal.mol^-1^ order. In contrast, the D9-designed compound demonstrates the minimized energy in kcal.mol^-1^ order, as displayed in Table 6, highlighting its remarkable stability and strong binding to the NMDA receptor, all compared to the other designed compounds.

**Table 6:** Bonding energies, Cartesian coordinates of Grid box, and produced interactions for the five studied complexes.

Complex	Binding energy	Grid box	Intermolecular interactions
(D9-NMDAR)	−7.76	X = 83.161 Y = 11.011 Z = −37.326	Tyr109- Phe114-Ala75-Ile111-Gln110
(D10-NMDAR)	−10.36		Tyr109-Asp138-Ala71-Thr103-Thr105-Pro106-Leu102
(D11-NMDAR)	−8.53		Tyr109-Asp138-Gln118-Phe114-Ala71-Pro106-Thr105-Thr103
(D12-NMDAR)	−7.19		Tyr109-Asp138-Asp136-Gln118-Ala71
(THA drug-NMDAR)	−7.65		Tyr109- Ile111-Phe114-Pro78

### 3.5. Validation procedure for molecular docking

**Fig 10:**
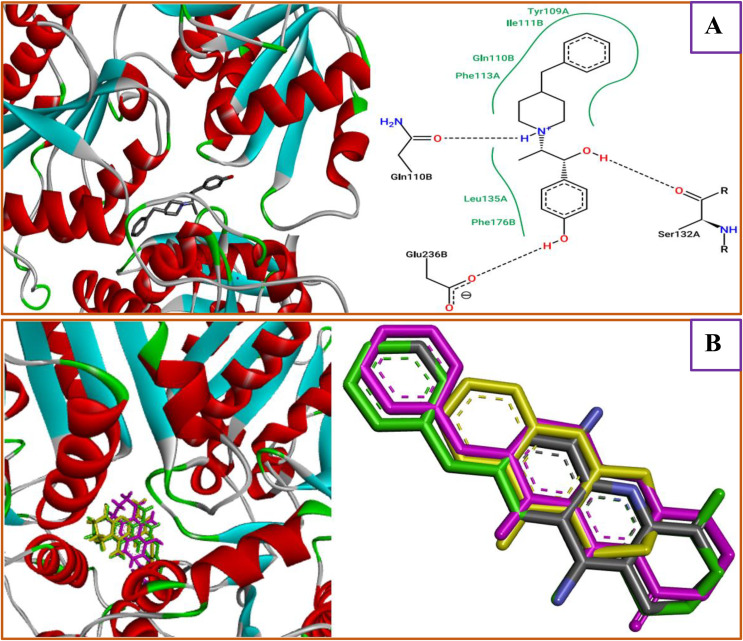
(A) Active binding sites of ifenprodil co-crystallized ligand on NMDAR, and (B) Alignment result of D9, D10, D11, and D12 on THA drug.

To examine the efficiency of the molecular docking simulations, the binding interactions produced for the THA drug and four designed PhO-THA analogs within the NMDA receptor (https://www.rcsb.org/structure/5EWJ) were first compared with the experimentally determined active site defined by the co-crystallized native ligand, Ifenprodil, a highly selective and non-competitive NMDA receptor antagonist that specifically targets the GluN2B subunit (Fig 10).

Among known NMDA receptor ligands, ifenprodil and its phenylethanolamine-based derivatives represent an important class of synthetic compounds that act as highly selective, non-competitive antagonists of GluN2B-containing NMDARs. These receptors are critically involved in normal physiological processes, including neuronal development, synaptic plasticity, learning, and memory. Importantly, GluN2B-selective antagonists have shown encouraging results in several clinical trials and display a more favorable side-effect profile compared with non-selective NMDA antagonists such as ketamine [36].

Analysis via the Protein-Plus server identified three key amino acid residues (AARs) within the 5EWJ.pdb structure: Ser132 and Glu236 in A chain, and Gln110 in B chain, as presented in the Fig 10A. These pivotal residues constitute the essential active sites for Ifenprodil recognition, serving as the standard for verifying the binding orientation of the newly designed PhO-THA analogues. For comparison, a common interaction was detected for the D9-designed compound, which was complexed to the same Gln110 active site, confirming that the candidate ligand has not been docked away from the NMDA receptor active sites. Additionally, D9, D10, D11, D12, and THA drug share similar intermolecular interactions such as Gln110, Ser132, Glu236, Phe114, and Tyr109 which were equally detected for the same targeted receptor (PDB ID of 5EWJ) towards the commercial brominated diphenyl ether (BDE) congeners, which were identified as persistent organic pollutants due to their ability to travel long distances in the environment, posing significant environmental threats and serious risks to human health, disrupting the GluN2B-containing NMDA receptors [37].

Thereafter, the root mean square deviations were well detected using the AutoDockTools-1–5.7rc1 application. The superposition method (Fig 10B) revealed that the four compounds D9, D10, D11, and D12 were superimposed on the THA drug with minimal root-mean-square deviations, which did not exceed 3Å, as presented in Table 7, which confirms again that the validation of the docking protocol was achieved successfully.

**Table 7:** RMSD values resulted from the superposition of D9, D10, D11, and D12 on THA drug.

Superposition result on THA drug	D9	D10	D11	D12
RMSD values	2.677	0.125	0.187	1.209

### 3.6. MD simulations

**Fig 11:**
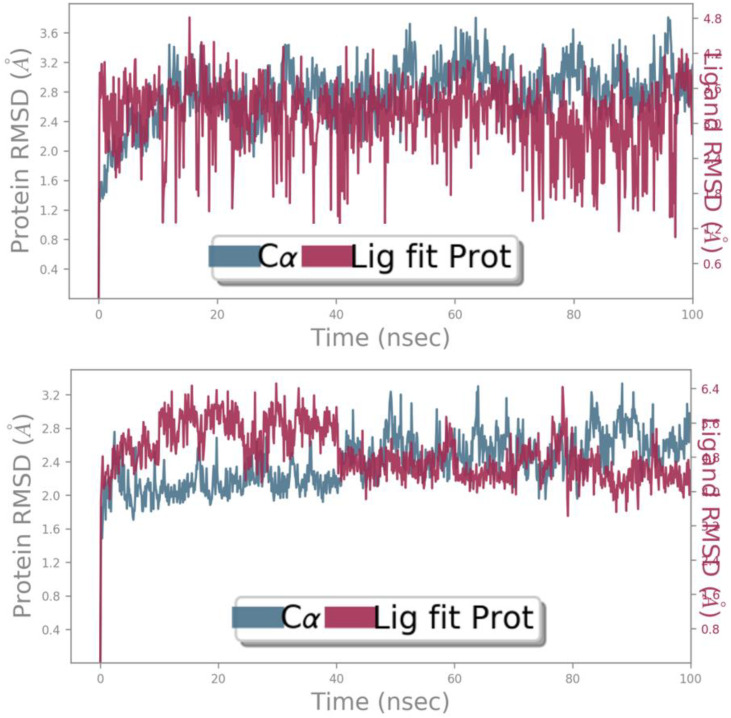
RMSD profiles showing conformational changes of the NMDA receptor over 100 ns in complexes with the THA drug and the designed compound D9, respectively.

**Fig 12:**
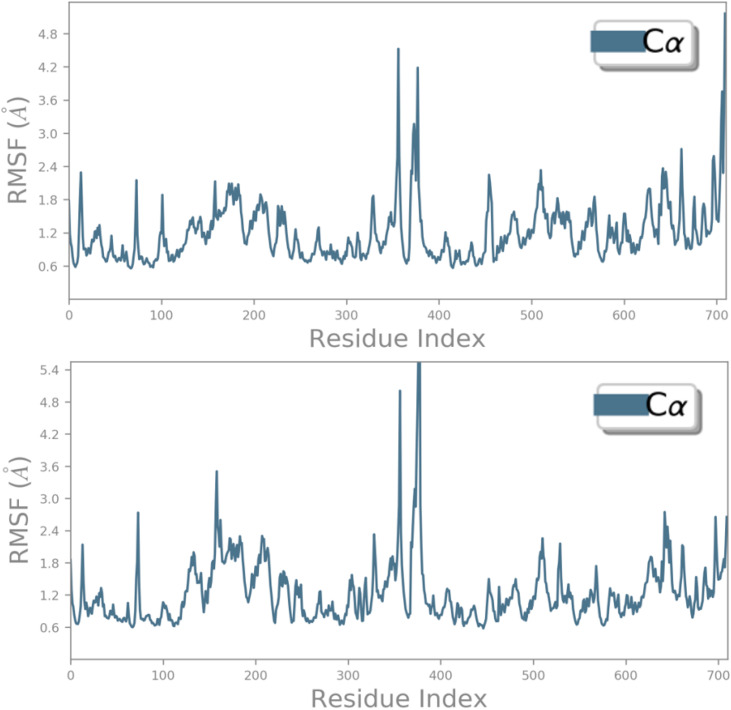
RMSF profiles showing conformational changes of the NMDA receptor over 100 ns in complexes with the THA drug and the designed compound D9, respectively.

**Fig 13:**
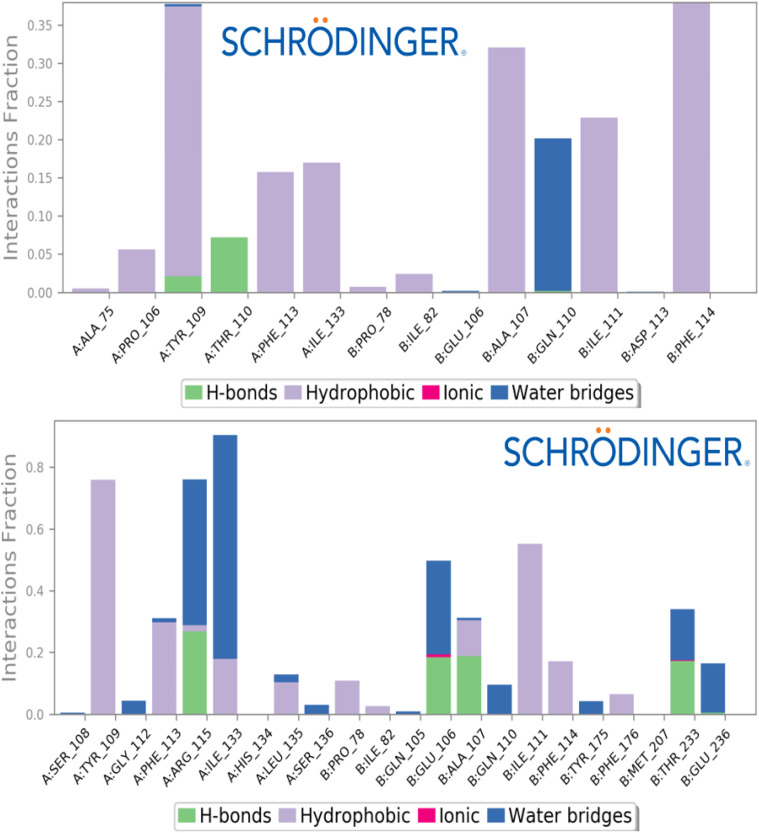
Protein-ligand contacts with interactive fractions for the NMDA receptor in complex with THA drug and D9 designed compound, respectively.

To assess the thermodynamic stability at the molecular level of the intermolecular interactions previously identified through molecular docking for both the reference drug and the designed ligand D9, free energy landscape (FEL) analysis was performed on the MD simulation results. Key parameters, including root mean square deviations (RMSD), root mean square fluctuations (RMSF), principal component analysis (PCA), and solvent-accessible surface area (SASA) were evaluated, confirming a high degree of molecular stability for both compounds when bound to the NMDA receptor. The thermodynamic stability of the macromolecular complexes, comprising the designed ligand and the reference ligand in association with the NMDA receptor, was supported by minimal conformational changes, which remained close to equilibrium and did not exceed a 3 Å deviation over the 100 ns MD simulation. RMSD analysis of both complexes revealed that the designed ligand D9 maintained stable behavior, with the Cα backbone RMSD ranging from 1.8 to 3.0 Å, while the ligand itself fluctuated between 4.2 and 5.6 Å values closely matching those observed for the standard drug THA (Fig 11). These results indicate strong and consistent binding of both candidate ligands to the NMDA receptor. Similarly, RMSF analysis showed nearly identical conformational flexibility of the NMDA receptor residues upon complexation with both THA and D9. The Cα backbone RMSF values mostly fell within 0.6–2.4 Å, aside from some terminal residues, reflecting high structural stability comparable to that of the reference drug. Fig 12 illustrates the RMSF profiles, highlighting minimal fluctuations over the 100 ns MD simulation period. Furthermore, analysis of protein-ligand contacts via histograms confirmed that the intermolecular interactions identified during docking, particularly those involving Tyr109, Phe114, and Ile111, play a critical role in maintaining the observed stability (Fig 13).

SASA analysis during the MD simulation reveals the receptor stability as well as ligand binding by assessing the buried and exposed hydrophobic regions in the macromolecular complexes. The SASA analysis for the concerned macromolecular complex of the NMDA receptor and the designed D9 is fluctuating within the range of 20–75 Å^2^, in the increasing pattern of SASA with simulation time, indicating the conformational change of the target receptor because of the binding of the designed ligand D9 in an identical manner to that of the standard drug THA. Radius of Gyration (Rg) correlates the compactness and stability of a drug-receptor complex over time to give insights into conformational changes, folding behavior, and binding effects. Decline in Rg post ligand binding suggests a more compact and stable complex, and the same was detected in the case of NMDAR complexed with designed molecule D9, as its initial Rg was 4.2 Å, which declined to 3.9 Å till the end of the simulation. More details are provided in S1 File and S2 File in S1 Data.

## 4. Conclusions

In conclusion, the present work highlighted a successful design of four safe compounds, labeled D9, D10, D11, and D12, thanks to two robust and highly validated 3D-QSAR models. All four designed compounds were predicted as novel phenoxy tacrine derivatives with desired ADME-Tox pharmacokinetic and physicochemical profiles. Based on both the findings of CoMSIA/HDA and CoMFA, the designed D9 was discovered to have minimal cytotoxic activity against Alzheimer’s disease, much closer to the efficacy of tacrine, making it a potential candidate drug simulated with excellent thermodynamic stability toward the NMDA receptor active sites over 100 nanoseconds of MD simulation time, similar to the tacrine drug. Therefore, the designed compound D9 is expected to have a promising role as an anti-Alzheimer’s agent, with high similarity to existing drug candidates. However, further clinical trials are highly recommended for greater credibility.

## Supporting information

S1 Data S1 File. Molecular dynamics simulation outputs for the designed compound D9. S2 File. Molecular dynamics simulation outputs for the reference drug THA.
(RAR)

## References

[1] Guo T, Zhang D, Zeng Y, Huang TY, Xu H, Zhao Y. Molecular and cellular mechanisms underlying the pathogenesis of Alzheimer’s disease. Mol Neurodegener. 2020;15(1):40. doi: 10.1186/s13024-020-00391-7 32677986 PMC7364557

[2] Crismon ML. Tacrine: first drug approved for Alzheimer’s disease. Ann Pharmacother. 1994;28(6):744–51. doi: 10.1177/106002809402800612 7919566

[3] Dogga B, Reddy EK, Sharanya CS, Abhithaj J, Arun KG, Ananda Kumar CS, et al. Design, synthesis and SAR studies of novel tacrine derivatives as potent cholinesterase inhibitors. European Journal of Medicinal Chemistry Reports. 2022;6:100094. doi: 10.1016/j.ejmcr.2022.100094

[4] Gniazdowska E, Koźmiński P, Halik P, Bajda M, Czarnecka K, Mikiciuk-Olasik E, et al. Synthesis, physicochemical and biological evaluation of tacrine derivative labeled with technetium-99m and gallium-68 as a prospective diagnostic tool for early diagnosis of Alzheimer’s disease. Bioorg Chem. 2019;91:103136. doi: 10.1016/j.bioorg.2019.103136 31374521

[5] Hamulakova S, Janovec L, Hrabinova M, Spilovska K, Korabecny J, Kristian P, et al. Synthesis and biological evaluation of novel tacrine derivatives and tacrine-coumarin hybrids as cholinesterase inhibitors. J Med Chem. 2014;57(16):7073–84. doi: 10.1021/jm5008648 25089370

[6] El fadili M, Er-rajy M, Abdalla M, Abuelizz HA, Zarougui S, Alkhulaifi FM, et al. In-silico investigations of novel tacrine derivatives potency against Alzheimer’s disease. Scientific African. 2024;23:e02048. doi: 10.1016/j.sciaf.2023.e02048

[7] El Fadili M, Er-Rajy M, Kara M, Assouguem A, Belhassan A, Alotaibi A, et al. QSAR, ADMET In Silico Pharmacokinetics, Molecular Docking and Molecular Dynamics Studies of Novel Bicyclo (Aryl Methyl) Benzamides as Potent GlyT1 Inhibitors for the Treatment of Schizophrenia. Pharmaceuticals (Basel). 2022;15(6):670. doi: 10.3390/ph15060670 35745588 PMC9228289

[8] Olivares D, Deshpande VK, Shi Y, Lahiri DK, Greig NH, Rogers JT, et al. N-methyl D-aspartate (NMDA) receptor antagonists and memantine treatment for Alzheimer’s disease, vascular dementia and Parkinson’s disease. Curr Alzheimer Res. 2012;9(6):746–58. doi: 10.2174/156720512801322564 21875407 PMC5002349

[9] Rosini M, Simoni E, Minarini A, Melchiorre C. Multi-target design strategies in the context of Alzheimer’s disease: Acetylcholinesterase inhibition and NMDA receptor antagonism as the driving forces. Neurochem Res. 2014;39(10):1914–23. doi: 10.1007/s11064-014-1250-1 24493627

[10] Gorecki L, Misiachna A, Damborsky J, Dolezal R, Korabecny J, Cejkova L, et al. Structure-activity relationships of dually-acting acetylcholinesterase inhibitors derived from tacrine on N-methyl-d-Aspartate receptors. Eur J Med Chem. 2021;219:113434. doi: 10.1016/j.ejmech.2021.113434 33892271

[11] Kaniakova M, Korabecny J, Holubova K, Kleteckova L, Chvojkova M, Hakenova K, et al. 7-phenoxytacrine is a dually acting drug with neuroprotective efficacy in vivo. Biochemical Pharmacology. 2021;186:114460. doi: 10.1016/j.bcp.2021.114460 33571502

[12] El fadili M, Er-rajy M, Imtara H, Kara M, Zarougui S, Altwaijry N, et al. 3D-QSAR, ADME-Tox in silico prediction and molecular docking studies for modeling the analgesic activity against neuropathic pain of Novel NR2B-Selective NMDA receptor antagonists. Processes. 2022;10(8):1462. doi: 10.3390/pr10081462

[13] Er-Rajy M, El Fadili M, Mujwar S, Zarougui S, Elhallaoui M. Design of novel anti-cancer drugs targeting TRKs inhibitors based 3D QSAR, molecular docking and molecular dynamics simulation. J Biomol Struct Dyn. 2023;41(21):11657–70. doi: 10.1080/07391102.2023.2170471 36695085

[14] Zadorozhnii PV, Kiselev VV, Kharchenko AV. In Silico ADME profiling of salubrinal and its analogues. Future Pharmacology. 2022;2(2):160–97. doi: 10.3390/futurepharmacol2020013

[15] Aloui M, Er-Rajy M, Imtara H, Goudzal A, Zarougui S, El Fadili M, et al. QSAR modelling, molecular docking, molecular dynamic and ADMET prediction of pyrrolopyrimidine derivatives as novel Bruton’s tyrosine kinase (BTK) inhibitors. Saudi Pharm J. 2024;32(1):101911. doi: 10.1016/j.jsps.2023.101911 38226346 PMC10788635

[16] Ed‐Dahmani I, El fadili M, Nouioura G, El Atki Y, Kandsi F, Conte R, et al. Chemical composition, antioxidant properties, acute toxicity, and pharmacokinetic evaluation of aqueous extract of roots of Ferula communis L. ChemistrySelect. 2024;9(43). doi: 10.1002/slct.202403973

[17] Er-rahmani S, El fadili M, Trotta F, Matencio A, Errabiti B, Abed SE, et al. Antimicrobial and antiadhesive activities of secondary metabolites against Bacillus cereus adhesion on PLA 3D printing material: ADMET Tox in silico, molecular docking and molecular dynamic analysis. Scientific African. 2024;24:e02209. doi: 10.1016/j.sciaf.2024.e02209

[18] Jung D, Floyd J, Gund TM. A comparative molecular field analysis (CoMFA) study using semiempirical, density functional, ab initio methods and pharmacophore derivation using DISCOtech on sigma 1 ligands. J Comput Chem. 2004;25(11):1385–99. doi: 10.1002/jcc.10410 15185333

[19] Klebe G, Abraham U, Mietzner T. Molecular similarity indices in a comparative analysis (CoMSIA) of drug molecules to correlate and predict their biological activity. J Med Chem. 1994;37(24):4130–46. doi: 10.1021/jm00050a010 7990113

[20] El Fadili M, Ez-Zoubi A, Aloui M, Mujwar S, Abuelizz HA, Elhalaoui M, et al. Design of novel pyrazole and benzofuran-based derivatives as potent acetylcholinesterase inhibitors for Alzheimer’s disease management. Front Chem. 2025;13:1614462. doi: 10.3389/fchem.2025.1614462 40475253 PMC12137266

[21] Er-rajy M, El fadili M, Mujwar S, Imtara H, Al kamaly O, Zuhair Alshawwa S, et al. Design of novel anti-cancer agents targeting COX-2 inhibitors based on computational studies. Arabian Journal of Chemistry. 2023;16(10):105193. doi: 10.1016/j.arabjc.2023.105193

[22] Ed-Dahmani I, El Fadili M, Kandsi F, Conte R, El Atki Y, Kara M, et al. Phytochemical, antioxidant activity, and toxicity of wild medicinal plant of melitotus albus extracts, in vitro and in silico approaches. ACS Omega. 2024;9(8):9236–46. doi: 10.1021/acsomega.3c08314 38434823 PMC10905593

[23] Nouioura G, El Fadili M, El Barnossi A, Loukili EH, Laaroussi H, Bouhrim M, et al. Comprehensive analysis of different solvent extracts of Ferula communis L. fruit reveals phenolic compounds and their biological properties via in vitro and in silico assays. Sci Rep. 2024;14(1):8325. doi: 10.1038/s41598-024-59087-3 38594363 PMC11004150

[24] Ed-Dahmani I, El Fadili M, Nouioura G, Kandsi F, Atki YE, Abuelizz HA, et al. Ferula communis leaf extract: antioxidant capacity, UHPLC-MS/MS analysis, and in vivo and in silico toxicity investigations. Front Chem. 2025;12:1485463. doi: 10.3389/fchem.2024.1485463 39925381 PMC11803407

[25] Misiachna A, Svobodova B, Netolicky J, Chvojkova M, Kleteckova L, Prchal L, et al. Phenoxytacrine derivatives: Low-toxicity neuroprotectants exerting affinity to ifenprodil-binding site and cholinesterase inhibition. Eur J Med Chem. 2024;266:116130. doi: 10.1016/j.ejmech.2024.116130 38218127

[26] El fadili M, Er-rajy M, Ali Eltayb W, Kara M, Assouguem A, Saleh A, et al. In-silico screening based on molecular simulations of 3,4-disubstituted pyrrolidine sulfonamides as selective and competitive GlyT1 inhibitors. Arabian Journal of Chemistry. 2023;16(10):105105. doi: 10.1016/j.arabjc.2023.105105

[27] El Fadili M, Er-Rajy M, Imtara H, Noman OM, Mothana RA, Abdullah S, et al. QSAR, ADME-Tox, molecular docking and molecular dynamics simulations of novel selective glycine transporter type 1 inhibitors with memory enhancing properties. Heliyon. 2023;9(2):e13706. doi: 10.1016/j.heliyon.2023.e13706 36865465 PMC9971180

[28] Nouioura G, El fadili M, Ghneim HK, Zbadi L, Maache S, Zouirech O, et al. Exploring the essence of celery seeds (Apium graveolens L.): Innovations in microwave-assisted hydrodistillation for essential oil extraction using in vitro, in vivo and in silico studies. Arabian Journal of Chemistry. 2024;17(5):105726. doi: 10.1016/j.arabjc.2024.105726

[29] El Fadili M, Er-Rajy M, Ali Eltayb W, Kara M, Imtara H, Zarougui S, et al. An in-silico investigation based on molecular simulations of novel and potential brain-penetrant GluN2B NMDA receptor antagonists as anti-stroke therapeutic agents. J Biomol Struct Dyn. 2024;42(12):6174–88. doi: 10.1080/07391102.2023.2232024 37428078

[30] El Fadili M, Er-Rajy M, Mujwar S, Ajala A, Bouzammit R, Kara M, et al. In silico insights into the design of novel NR2B-selective NMDA receptor antagonists: QSAR modeling, ADME-toxicity predictions, molecular docking, and molecular dynamics investigations. BMC Chem. 2024;18(1):142. doi: 10.1186/s13065-024-01248-6 39085870 PMC11293250

[31] Kciuk M, Mujwar S, Rani I, Munjal K, Gielecińska A, Kontek R, et al. Computational Bioprospecting Guggulsterone against ADP Ribose Phosphatase of SARS-CoV-2. Molecules. 2022;27(23):8287. doi: 10.3390/molecules27238287 36500379 PMC9739500

[32] Mujwar S. Computational bioprospecting of andrographolide derivatives as potent cyclooxygenase-2 inhibitors. Biomedical and Biotechnology Research Journal (BBRJ). 2021;5(4):446–50. doi: 10.4103/bbrj.bbrj_56_21

[33] Mujwar S, Harwansh RK. In silico bioprospecting of taraxerol as a main protease inhibitor of SARS-CoV-2 to develop therapy against COVID-19. Struct Chem. 2022;33(5):1517–28. doi: 10.1007/s11224-022-01943-x 35502321 PMC9046011

[34] Kciuk M, Mujwar S, Szymanowska A, Marciniak B, Bukowski K, Mojzych M, et al. Preparation of Novel Pyrazolo[4,3-e]tetrazolo[1,5-b][1,2,4]triazine Sulfonamides and Their Experimental and Computational Biological Studies. Int J Mol Sci. 2022;23(11):5892. doi: 10.3390/ijms23115892 35682571 PMC9180621

[35] Bouzammit R, Lakkab I, El fadili M, Kanzouai Y, Chalkha M, Nakkabi A, et al. Synthesis, crystal structure, antioxidant activity and molecular docking studies of 2-(1-(3-methyl-1-oxo-1,2,3,4-tetrahydronaphthalen-2-yl)ethyl)malononitrile. Journal of Molecular Structure. 2024;1312:138582. doi: 10.1016/j.molstruc.2024.138582

[36] Stroebel D, Buhl DL, Knafels JD, Chanda PK, Green M, Sciabola S, et al. A novel binding mode reveals two distinct classes of NMDA Receptor GluN2B-selective Antagonists. Mol Pharmacol. 2016;89(5):541–51. doi: 10.1124/mol.115.103036 26912815 PMC4859819

[37] Karakuş F, Kuzu B. Mechanistic analysis of decabromodiphenyl ether-induced neurotoxicity in humans using network toxicology and molecular docking. Neurotox Res. 2025;43(2):17. doi: 10.1007/s12640-025-00741-7 40123016 PMC11930881

